# Supplementary material for: Assembling of Metal-Polymer Nanocomposites in Irradiated Solutions of 1-Vinyl-1,2,4-triazole and Au(III) Ions: Features of Polymerization and Nanoparticles Formation
Source: Polymers (Basel). 2022 Oct 29;14(21):4601. doi: 10.3390/polym14214601 (PMC9657828; doi:10.3390/polym14214601)
Supplement: Supplementary file 1 [file polymers-14-04601-s001.zip › polymers-1988864-supplementary.pdf]

Assembling of Metal-Polymer Nanocomposites in Irradiated Solutions of 1-Vinyl-1,2,4-triazole and Au(III) Ions: Features of Polymerization and Nanoparticles Formation

Alexey A. Zharikov [1], Elena A. Zezina [1], Rodion A. Vinogradov [1], Alexander S. Pozdnyakov [2], Vladimir I. Feldman [1], Sergey N. Chvalun [3,*], Alexander L. Vasiliev [4] and Alexey A. Zezin [1,3]

[1] Department of Chemistry, Lomonosov Moscow State University, 119991, Moscow, Russia
[2] Favorsky Irkutsk Institute of Chemistry, Siberian Branch of the Russian Academy of Sciences, Favorsky st., 1, 664033, Irkutsk, Russia
[3] Enikolopov Institute of Synthetic Polymeric Materials, Russian Academy of Sciences, 70, Profsoyuznaya st., 70, 117393, Moscow, Russia
[4] Moscow Institute of Physics and Technology (National Research University), 141701, Dolgoprudny, Moscow Region, Russia
* s-chvalun@yandex.ru.

Supplementary Materials

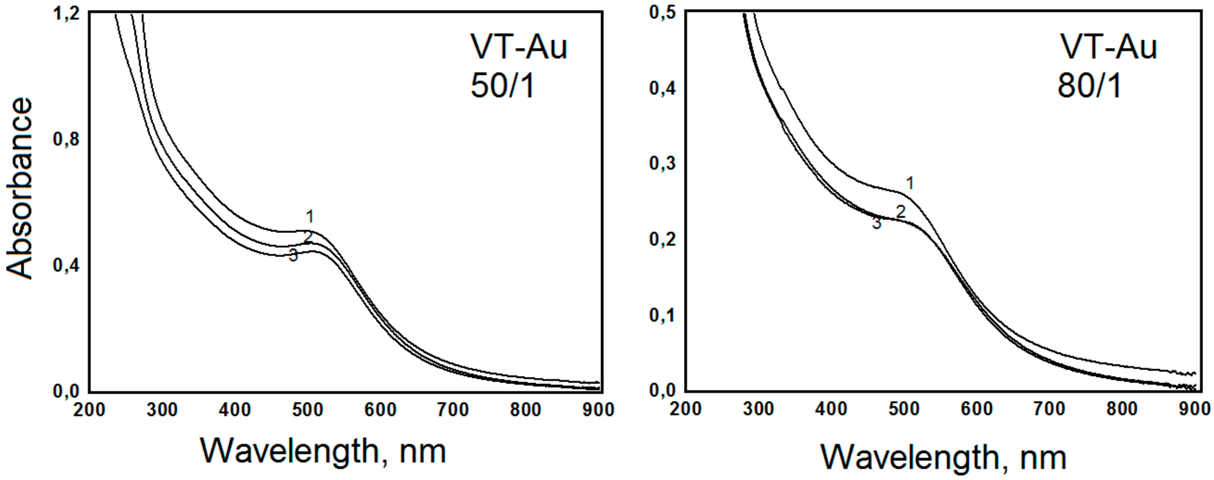

Figure S1. The UV-VIS spectra of irradiated at pH 5.5 VT-Au(III) solutions (1 - the spectrum was recorded immediately after irradiation) and the spectra of Au-colloids after 15 hours (curve 2) and 5 days (curve 3).
